# Genetic Diversity of Human Enterovirus in Kazakhstan, during 2022

**DOI:** 10.1155/2024/7796913

**Published:** 2024-08-26

**Authors:** Dinara Kamalova, Assel Akhmetova, Asylulan Amirgazin, Igor Sytnik, Viktoriya Rudenko, Gulzhan Yessimkhanova, Dinagul Bayesheva, Sergey Yegorov, Alexander Shevtsov

**Affiliations:** ^1^ National Center for Biotechnology, Astana, Kazakhstan; ^2^ L. N. Gumilyov Eurasian National University, Astana, Kazakhstan; ^3^ Astana Medical University, Astana, Kazakhstan; ^4^ Department of Biology School of Sciences and Humanities Nazarbayev University, Astana, Kazakhstan

## Abstract

Enteroviral infection is a common cause of aseptic meningitis, herpangina, and hand, foot, and mouth disease in children. Limited data are available on the enteroviral subtypes associated with hospitalization for these conditions in Kazakhstan. We collected cerebrospinal fluid (CSF) and nasopharyngeal swabs (NSW) from children (*N* = 152, median age = 8 years) hospitalized with symptoms of aseptic meningitis (AM, *N* = 139) or herpangina (HA, *N* = 13) disease. We then genotyped enteroviral subtypes associated with AM (*n* = 50) and HA (*n* = 9) using next-generation sequencing (NGS) on the viral protein 1 (VP1), followed up by whole-genome sequencing of the isolated viral species. All identified EVs were species B EV, consisting of five echoviruses (E6, E9, E11, E21, and E25) and three coxsackieviruses (CVA9, CVB3, and CVB5) serotypes within the cohort. The most abundant EVs were CVA9 (38.5%), CVB5 (21.5%), and E6 (13.8%). Most HA samples (6/9) were genotyped with coxsackievirus CVA9, while AM was associated with a variety of both echovirus and coxsackievirus serotypes. The results suggest that coxsackievirus CVA9 may be the dominant serotype circulating in the HA population, while AM is more diverse in terms of circulating echovirus and coxsackievirus serotypes. Further studies are needed to determine the clinical implications of these findings and to investigate potential differences in disease severity or outcomes associated with different EV serotypes.

## 1. Introduction

Human enteroviruses (HEVs) are small single stranded RNA viruses of the family *Picornaviridae* (https://www.picornaviridae.com) that cause different types of infectious illnesses in humans affecting millions of people every year in the world. Symptoms can vary from mild to severe and can result in syndromes like respiratory diseases, herpangina, hand-foot-and-mouth disease, aseptic meningitis, acute hemorrhagic conjunctivitis, and myocarditis. [1, 2]. Herpangina and meningitis in children are significant illnesses caused by enteroviruses because of the potential severity and impact on children's health. Herpangina is highly contagious and spread very quickly, and it mostly affects younger children and is characterized by fever, sore throat, and small ulcers in the mouth and at the back of the throat [3, 4]. The ulcers can be painful and cause discomfort for children when drink and eat, which increases the risk of dehydration [3]. Symptoms can be confused with other illnesses; therefore, accurate diagnosis is significant for proper management and treatment [1, 4]. Herpangina complications are rare; however, in some cases, they can cause brainstem encephalitis and acute flaccid paralysis.

Based on serological tests, enteroviruses (EVs) were originally divided into coxsackieviruses A and B, echoviruses, and polioviruses subgroups [5]. Species of the genus Enterovirus are divided based on their pathogenicity, to human (Enterovirus A–D and Rhinovirus A–C) and animal species (Enterovirus E–J) [6]. However, with the development of molecular biology methods, classification is based on genetic characteristics and phylogenetic relationships between strains [7], where each EV species includes different serotype groups. Molecular typing of HEV serotypes is important for many reasons, such as in case of severe clinical disorders (some serotypes can cause fatal neurological disease), for the discrimination of polio and nonpolio enteroviruses, as well as for epidemiological and phylogenetic studies (discovering newly occurring serotypes) [8].

The EVs structure comprises nonenveloped positive stranded RNA molecule (approximately, 7400 nt long), which is surrounded by the structural proteins VP1 to VP4 (each in sixty copies) that form icosahedral capsid structure of the virus [9]. The genome of enterovirus is a single RNA molecule with small virus-encoded protein VPg at the 5′ end and untranslated region (UTR) from both 5′ and 3′ ends [10]. Many research studies have shown that VP1 capsid protein is a good marker for enterovirus serotypes' differentiation and is widely used for PCR-based typing [10–12]. Recently known detection and classification methods are based on reverse-transcription polymerase chain reaction (RT-PCR) [13].

The HEV-B species consists of the vast majority of EV serotypes, and according to King's [14] virus taxonomy, it includes coxsackievirus A9 (CVA9), coxsackieviruses B (CVB) 1–6, echoviruses (EV) 1–7, 9, 11–21, 24–27, 29–33, and enterovirus 69. Moreover, only coxsackievirus A9 from all coxsackieviruses belongs to HEV-B, while others belong to HEV-A and HEV-C [14].

Enteroviruses can be transmitted among humans in different ways: direct contact (fecal-oral way) and indirect contact (contaminated equipment) or drinking water from infected reservoirs. Infections predominate in the summer and autumn seasons, however, can occur sporadically during the year. According to previous research, younger children are known to be affected more often comparing to adults [15, 16]. Although, in most cases, infections caused by EVs occur with mild symptoms as respiratory or gastrointestinal illnesses, some of them can cause severe complications for patients, from central nervous system invasion to meningitis, encephalitis, and paralysis [17].

Understanding the molecular signatures of HEVs is essential for effective diagnosis and treatment protocols. The situation with HEV infection molecular typing in Kazakhstan is not yet fully studied, and there is no whole genome data available yet. This research study describes clinical diseases and associated HEV serotypes, as well as whole genome sequencing of 65 EV isolates collected from children in central infectious diseases hospital, located in Astana in 2022. The aim of the study is to investigate the molecular epidemiology of occurring HEV infection and the prevalence of circulating serotypes in Kazakhstan. In this study, we analyzed 65 sequences of HEV and compared with the publicly available NCBI database to understand important evolutionary dynamics and infection spread.

## 2. Materials and Methods

### 2.1. Patients and Selection Criteria

This study provides a retrospective analysis of 152 cases of hospitalization of children in the 3rd Children's Infectious Diseases Hospital in Astana from May 14 to October 12, 2022, with a preliminary diagnosis of enterovirus infection. Children's Infectious Diseases Hospital Astana is the only specialized institution providing inpatient treatment for childhood infections in Astana (population 1.35 million people).

Enterovirus infections were classified by clinical phenotypes, using the following definitions: herpangina (HA) and aseptic meningitis (AM) caused by enterovirus.

Specimens for the current study were collected, stored, and transported according to the CDC guidelines (https://www.cdc.gov/non-polio-enterovirus/lab-testing/specimen-collection.html). Patient specimens for “rapid” molecular diagnostics were processed in the rapid laboratory, while samples for “delayed” molecular diagnostics and “routine” diagnostics were stored at −20°C.

1. Nasopharyngeal and nasal swabs were collected within the first three days of illness. Swabs were collected using a dry, sterile probe with a cotton swab, which, after collection, was placed in a disposable tube containing 500 *μ*l of transport medium (0.9% sodium chloride solution or phosphate buffer).
2. Cerebrospinal fluid (CSF) was collected under aseptic conditions during the first days of the disease when clinical indications for lumbar puncture were present. A volume of 1 ml was collected in a sterile disposable plastic tube.

Samples from patients selected for “rapid” molecular diagnostic group were processed in the rapid diagnostic laboratory as soon as they were received. Specimens for “delayed” molecular diagnostic testing from subjects randomized to the “conventional” diagnostic group were stored at −20°C. Delivery to the laboratory was made within 12 hours in thermal containers with refrigeration at a temperature not exceeding +4°C.

Herpangina and hand-foot-mouth diseases (HFMD) cause similar symptoms with ulcers in or around the mouth, with HFMD causing vesicular lesions on the arms, legs, knees, or buttocks. A nasopharyngeal swab (NSW) was taken from patients using a sterile cotton swab and placed in a transport medium (DMEM with 50 *μ*g/ml of gentamicin) and stored at minus 70°C until RNA extraction.

Aseptic meningitis was accompanied by an increase in body temperature up to 38−40°C, the development of neck rigidity, headaches, and photophobia was observed, and some patients experienced vomiting, loss of appetite, diarrhea, rash, pharyngitis, and myalgia. For patients with signs of meningitis, according to the standard protocol, a cerebrospinal fluid (CSF) puncture was prescribed, which was divided into 3 equal aliquots by 200 *μ*l. According to a standard diagnostic protocol, two CSF sample aliquots were used for enterovirus diagnosis. One sample was analyzed using bacteriology and cytology methods at the hospital laboratory. The second aliquot was sent for laboratory analysis using RT-PCR kit AmpliSens® Human Enterovirus-FL (AmpliSens, Russia) in the RSE “Center for Sanitary and Epidemiological Expertise” Medical Centre Hospital of President's affairs Administration of the Republic of Kazakhstan. The third part of the CSF was stored at minus 80°C for additional studies and genotyping. In case of presence of vesicular lesions of the oral cavity, a nasopharyngeal swab was also collected from patients.

In total, for the specified period, 139 children were hospitalized with aseptic meningitis diagnosis confirmed and the presence of enterovirus RNA in cerebrospinal fluid samples detected. Herpangina was diagnosed in 13 children patients, and enterovirus RNA detected from NSW samples.

### 2.2. RNA Extraction

Viral RNA was extracted from 200 *μ*l CSF or NSW samples in transport medium using GeneJET kit Viral DNA and RNA Purification Kit (ThermoScientific, Lithuania) according to the manufacturer's instructions.

### 2.3. Whole Genome Sequencing and Assembly of Genomes

Almost full-length genomes of enteroviruses were amplified as described previously by Isaacs et al. [18], except that the readily made reaction mix from BioMaster LR HS-PCR 2x (Biolabmix, Russia) was used for amplification step. Second-stage PCR products were purified with AMPure XP magnetic beads (Beckman Coulter, USA) in a 1 : 1 ratio, with elution in 20 *μ*l of DNase/RNase-free distilled water. Libraries were prepared using the Illumina® DNA Prep kit, (M) Tagmentation (96 Samples) (Illumina, Catalog #20018705) with double barcoding. Libraries were sequenced on Illumina MiSeq using MiSeq Reagent Kit v3 (600-cycle) (Catalog #LMS -102-3003). Reagents were used according to the manufacturers' instructions.

The quality control of the obtained reads was carried out using the FastQC program [19]. Before *denovo* assembly, reads were trimmed using SeqTK (with options: -b 20-e3) and sickle (with options: -t sanger-q 30-l 200-g), followed by deduplication in the FastP program (with options: -- dedup -- dup _ calc _ accuracy 6 -- disable _ quality _ filtering). To assemble the genomes, Megahit v1.2.9 was used with varying the length of *κ*-mers individually for each sample, selecting the assembly with the longest lengths for subsequent contigs analysis. The resulting contigs were then used as corresponding references to obtain consensus sequences using BWA. Variants were determined using FreeBayes, and consensuses were generated by BCFtools consensus. The ends of assemblies with less than 30x coverage were removed.

### 2.4. Determination of Serotypes

Serotypes were determined by constructing a phylogenetic tree from the database generated with VP1 sequences of enteroviruses from GenBank (https://www.ncbi.nlm.nih.gov/genbank/). The database was selected using keywords such as “enterovirus,” “VP1,” and “human enterovirus B.” In total, approximately 26,000 genome sequence data with VP1 regions were downloaded. Using the generated database and our sequences, a phylogenetic tree was reconstructed, which made it possible to filter the 81 isolates most closely related to our sequences from the database. Further analysis was performed with 146 enterovirus isolates, including 65 obtained within the current study, and Enterovirus 69 was selected as an outgroup (GenBank: AY302560.1) [20]. The statistical selection of the nucleotide substitution model for phylogenetic analysis was performed using ModelTest -NG (v0.1.7) [21], and the best fitting model was selected for the reconstruction of the phylogenies. Phylogenetic tree was built using the maximum likelihood (ML) method with the bootstrap random sampling method, *n* = 1000. Estimated bootstrap (bs) values greater than 50% were shown for the tree nodes. The Interactive Tree of Life (iTOL) [22] online tool was used for interpretation, visualization, and annotation of the phylogenetic tree.

### 2.5. Genotyping by VP1 Region

Representative datasets were downloaded separately for each serotype group to describe the molecular epidemiology of HEV-B serotypes in Kazakhstan. Phylogenetic relatedness of enterovirus isolates collected within the current study with the most recent genotyping and subgenotyping data was compared. Phylogenetic dendrograms were reconstructed based on entire VP1 sequences of the international strains downloaded from GenBank.

Genotyping of coxsackievirus A9 (CVA9) was based on Zhao et al. [23] algorithm, that included 110 international sequences and 25 obtained within the current study. Coxsackievirus B5 (CVB5) genotyping of 134 isolates, including 14 Kazakhstani, was performed using the same algorithm as described in He et al. [24]. Coxsackievirus B3 (CVB3) strains were analyzed based on Yang et al. [25], including 250 worldwide strains and 1 isolate from Kazakhstan. Genetic diversity of echoviruses group worldwide datasets and the relatedness of Kazakhstani isolates were reconstructed according to Cheng et al. [26] for Echovirus 6 (E6) (*n* = 73); Zhang et al. [27] for Echovirus 9 (E9) (*n* = 57); Li et al. [28] and Grapin et al. [29] for Echovirus 11 (E11) (*n* = 140). However, there are no currently available genotyping schemes for the Echovirus 21 (E21) and 25 (E25) groups. Therefore, entire VP1 coding sequences of available E21 and E25 serotypes were downloaded from GenBank (for August 15^th^, 2023) and phylogenetic dendrograms reconstructed, including sequences obtained within the current study. Genotypes were distinguished based on estimated divergence between/within groups, calculating pairwise genetic distances in MEGA X [30]. Genotypes determined with >15% difference between and <15% within groups.

The pairwise genetic distances for E21 and E25 serotypes were estimated within each serotype group using the Kimura 2-parameter model, with the gamma distribution model (shape parameter = 5) in MEGA X [30]. Enterovirus sequences were classified into so-called genotypes within each serotype (shown in Figures S1–S5) if they shared >85% sequence identity based within the VP1 capsid gene.

Sequences were aligned in Mafft (v7.520) [31], and the best-fitting substitution model was selected (for each serotype group) using the ModelTest-NG (v0.1.7) [21] program. Maximum likelihood tree reconstructed for VP1 sequences with bootstrapping of replicates 1000 times using RAxML-NG (v1.2.0). The ML tree was reconstructed to demonstrate the genetic diversity of the worldwide strains and the allocation of sequenced Kazakhstani enterovirus strains to particular genotype and subgenotype group. The Interactive Tree of Life (iTOL) (v1.0) [22] was used for illustration and annotation of the ML tree.

### 2.6. Analysis of Whole Genome Sequencing Data

Phylogenetic analysis of 65 whole genome sequences from 58 infected patients in Kazakhstan was performed. The nucleotide substitution model for evolutionary analysis was performed in ModelTest-NG (v0.1.7) [21], and the best-fitting model was selected as “GTR + I + G.” A phylogenetic tree was reconstructed using the maximum likelihood (ML) method with the bootstrap random sampling method in RAxML-NG (v1.2.0), with *n* = 1000 bootstraps. Statistically significant bootstrap (bs) values greater than 50% were shown for the tree nodes. To detect if a sufficient “temporal signal” was present in the data, TempEst (v1.5.3) [32] software was used. Phylogenetic tree was visualized and annotated in the Interactive Tree Of Life (iTOL) [22] online tool. Pairwise genetic distances were estimated using the *ape* package in R [33].

## 3. Results

### 3.1. Samples' Overview

**Figure 1:**
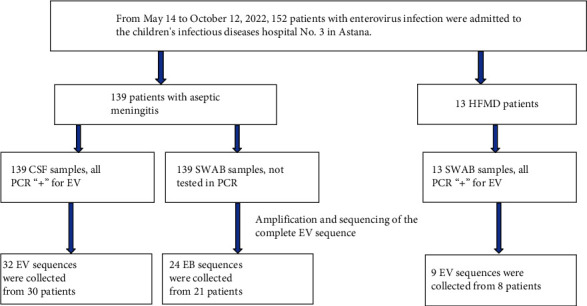
Flowchart showing the sampling scheme of the EV strains collected from infected patients in May-October 2022.

During the period from May 14 to October 12, 2022, 152 patients with a preliminary diagnosis of enterovirus infection were admitted to the Children's Infectious Diseases Hospital No. 3 in Astana. Schematic representation of the samples collected is shown in Figure 1.

**Figure 2:**
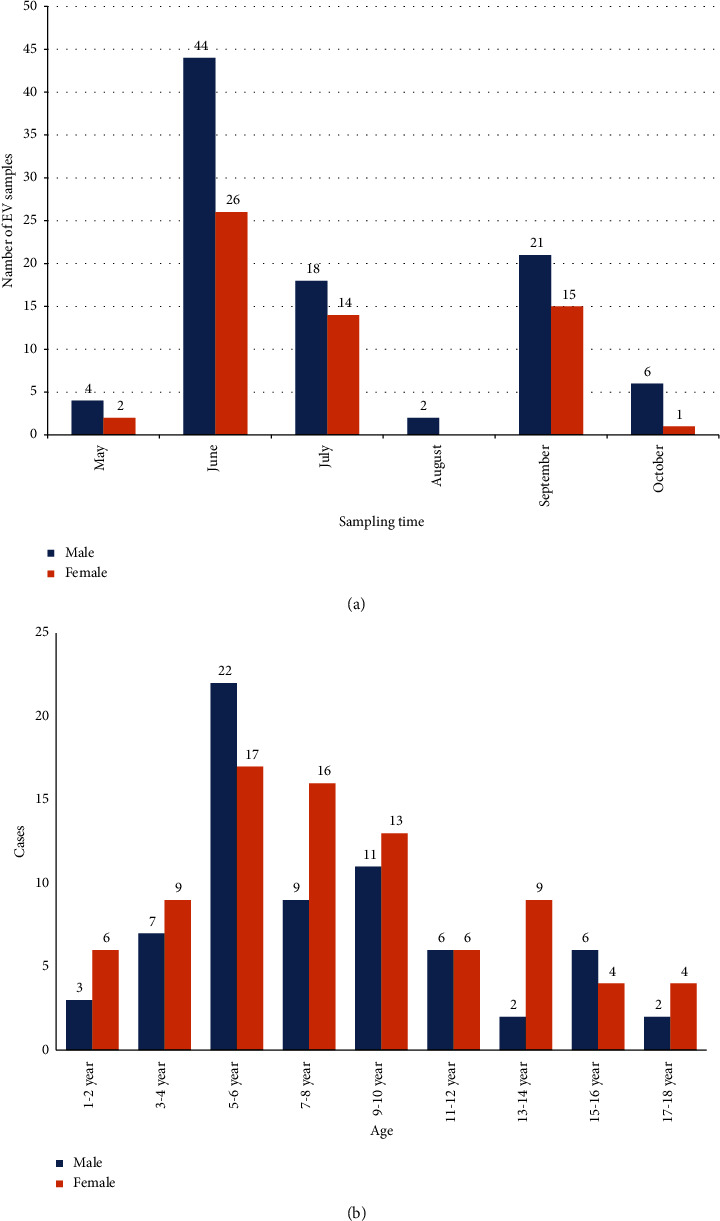
(a, b). Distribution of samples collected from patients for EV diagnosis: (a) by sampling time; (b) by age groups. *X*-axis represents number of samples collected from patients from total number of samples (*n* = 152). *Y*-axis shows (a) months; (b) age groups. Numbers on histogram bars show exact number of samples collected in female (orange)/male (blue) patients.

The largest number of patient visits occurred during the summer period, as shown in Figure 2; 67% of the studied patients were hospitalized in June-July. There was a decrease in the number of requests during August, followed by a peak increase in September (23.7%). The number of children under 10 years of age predominates and equals 74.3% (*n* = 113) of the total number of admitted patients, compared with the number of children aged over 10 years of 25.7% (*n* = 39) (average age of patients: 8.1 years). Moreover, among patients under the age of 10 years, more than 59% of patients were male (*n* = 67). Around 91.5% (139/152) of children were hospitalized with a diagnosis of aseptic meningitis and with a diagnosis of herpangina 8.5% (13/152).

Laboratory cytology results were available for 131 of 152 patients. The median value of blood leukocytes was 10.36 cells/L, with 25th and 75th percentile values of 7.99 cells/L and 13.27 cells/L, respectively. The median percentage of lymphocytes and neutrophils in CSF samples were 53% and 42%, respectively, with the values of the 25th and 75th percentiles being 20% and 77% for lymphocytes, and 22.5% and 79% for neutrophils, respectively.

## 4. Results of Whole-Genome Sequencing and Identification of Serotypes

Nearly complete whole-genome sequences of 65 enterovirus isolates were amplified and assembled from RNA samples collected from 58 patients. From all patients, 50 were diagnosed with aseptic meningitis and 8 with enterovirus infection herpangina. From 50 meningitis patients, 32 CSF samples and 24 NSW samples were selected for whole genome sequencing analysis. Nine sequences were obtained from NSW samples from 8 patients with enterovirus herpangina (Table 1).

**Figure 3:**
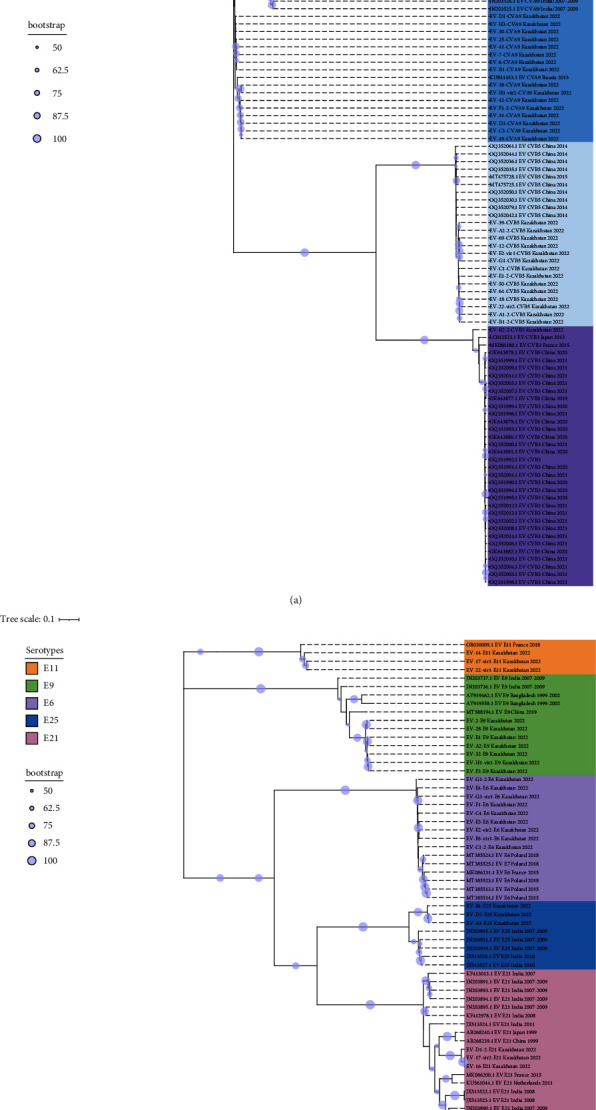
(a) Phylogenetic dendrograms were reconstructed by using the whole VP1 gene sequence of 65 human enterovirus B sequences collected from infected patients in Kazakhstan and 81 sequences downloaded from available worldwide human enterovirus B strains of serotypes CVA9, CVB3, and CVB5. (b) Phylogenetic dendrograms reconstructed by using the whole VP1 gene sequence of 65 human enterovirus B sequences collected from infected patients in Kazakhstan and 81 sequences downloaded from available worldwide human enterovirus B strains of serotypes E6, E9, E11, E21, and E25.

Consensus phylogenetic tree was built using the ML algorithm for the VP1 sequence region (Figures 3(a) and 3(b)). Phylogenetic clades were mostly separated by enterovirus serotypes, which is consistent with previous enterovirus research studies [34].

These strains were labeled using the following format: “GenBank accession number”/“Serotype”/“Country of origin”/“Year of isolation” (strains with partially available metadata were also included). The prototype Enterovirus B69 was used as an outgroup. Bootstrap values >50% are shown as blue circles.

**Figure 4:**
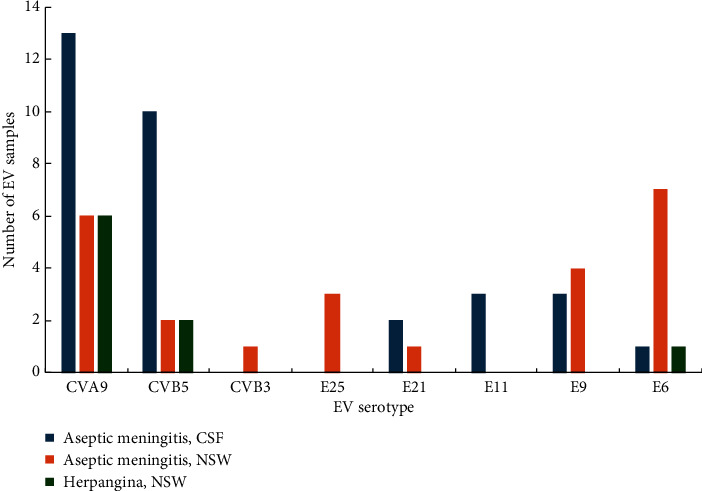
EV serotypes identified in patients with diagnosed aseptic meningitis disease and herpangina by sample types.

Based on phylogenetic analysis of the VP1 gene, 32 sequences obtained from CSF samples of 29 patients with meningitis were clustered into 6 serotypes: CVA9, CVB5, E6, E9, E11, and E21 (Figure 4). 24 sequences obtained from NSW samples of 21 patients with meningitis were clustered into 7 serotypes: CVA9, CVB5, CVB3, E6, E9, E21, and E25. Two CSF samples were found to be infected with both E21/CVB5 and E11/CVB5, respectively. In one patient, CVA9 was identified in a collected CSF sample and two serotypes were identified in the NSW sample (E9/CVA9) (Table 1).

Of the 9 sequences from 8 patients with enterovirus infection, 6 were identified as CVA9, 2 as CVB5, and 1 as E6, with one sample found to be coinfected with CVB5/E6 (Table 1, Figure 4).

### 4.1. Genotyping of Strains by the VP1 Gene

Subsequently, we analyzed genotypes based on the VP1 gene among certain serotypes. The obtained sequences were grouped with Asian and European genotype lineages (Figures S1–S8). The 25 coxsackievirus A9 samples clustered as a separate clade into genotype I and are genetically closest to the 2013 Russian sequence. 14 Coxsackievirus B5 sequences formed a cluster in genotype D and are genetically close to isolates circulating in China in 2015–2018. Nine echovirus 6 sequences cluster into genotype E and are genetically close to European and American lineages. Seven Echovirus 9 sequences cluster into a separate clade within genotype F, together with European lineages. Three Echovirus 11 sequences were included in the D5 subgenotype. This subgenotype also includes lineage 1 strains circulating in France and Spain in 2022 and 2023 and associated with neonatal infection with liver failure [29, 35, 36]. This lineage was clustered within a separate clade in subgenotype D5. A single coxsackievirus B3 sequence was clustered into the most prevalent genotype E.

For the analysis of Echovirus 21 genotypes, 31 complete VP1 sequences were downloaded from GenBank (sequences deposited until August 15, 2023). Current analysis included 3 sequences from Kazakhstan obtained as part of this study collected from CSF and NSW samples from patients with aseptic meningitis. Based on the phylogenetic dendrogram and percentage of identity between strains, E21 serotype can be divided into 10 genotypes A–J, the maximum difference in nucleotide sequences within one genotype was 12%, and the minimum average difference in nucleotide sequences between genotypes was 17% (Tables S1 and S2). Genotype A is represented by one echovirus 21 prototype strain Farina. Genotypes B, C, and F are represented by single sequences from Madagascar, China, and France. For genotypes D, E, and I, geographic relationships with strains from China, Central African Republic, and India are monitored. Genotype J combines strains from Asia and Europe. Three sequences from Kazakhstan were included in the H genotype, together with strains from China and the United Kingdom.

For the analysis of Echovirus 25 genotypes, 91 complete VP1 sequences were obtained from GenBank (sequences deposited until August 15, 2023). The analysis included 3 sequences from Kazakhstan obtained as part of this study. E25 can be divided into 8 genotypes A–H, and the identity of nucleotide sequences in the genotypes exceeded 85%. Kazakhstani strains are included in genotype A and are in a separate clade, which may subsequently become a subgenotype. Genotypes B and C include strains isolated from China and the USA, respectively. Genotypes D and H are the largest and combine strains circulating on the Eurasian and American continents. Genotype E combines two strains from the USA and Nigeria. Genotypes F and G include strains isolated in Europe.

### 4.2. Phylogenetic Analysis by Complete Coding Sequence

**Figure 5:**
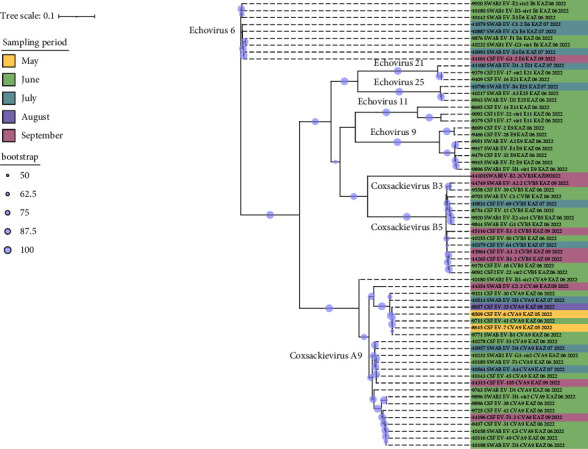
Phylogenetic analysis of the 65 complete coding sequences obtained in the current study.

Based on phylogenetic analysis of the complete coding sequence, 65 sequences were clustered according to specific serotypes (Figure 5).

The 25 CVA9 sequences formed three separate clusters, and the third cluster was represented by a single sequence. We did not find any features in the distribution of sequences in the clades in comparison with the sampling time. For example, sequences collected in June are evenly represented across all contributions, including a third clade represented by a single sequence from NSW from a patient with meningitis that is most genetically distant from all of them by over 3.7% (Table S3).

The CVA9 sequences of EV-H1-vir2 and EV-38 isolated from the same patient with CSF and NSW were 100% identical. The 14 CVB5 sequences formed two clusters, representing sequences collected from patients that were hospitalized in different periods. All 9 E9 sequences were collected in June and formed two clusters, differing from each other by 8% (Table S4).

The E6 sequences are the most genetically homogeneous, with less than 2% variability among themselves (Table S5).

## 5. Discussion

In Kazakhstan, cases of enterovirus infection are recorded annually, with peak values in the summer-autumn period [37]. In our study, more than 90% of hospitalized cases of enterovirus infection were due to aseptic meningitis, and only 8.5% were due to enterovirus herpangina. These data do not reflect the true picture of the spread of enterovirus HA in Kazakhstan since the classic mild course of herpangina may remain unreported due to home or local hospital treatment. The high number of cases of aseptic meningitis in our study correlates with previous study data, in which enteroviruses were identified as etiological agents in 73% of cases of encephalitis and meningitis in Kazakhstan, with an average incidence of 14 per 100,000 population, and for children under 15 years of age, the incidence is 35.9 per 100,000 children [38]. Despite the high incidence rate, information on circulating serotypes is limited. This is the first study to describe the genetic features of enteroviruses circulating in Kazakhstan based on nearly complete whole-genome data.

The highest incidence was recorded in the summer months, and more than 67% of patients were hospitalized in June-July months, followed by a decrease in hospitalization cases in August and a rise in incidence in September. Peaks of incidence in the summer-autumn period are typical for the northern hemisphere [39]. Of the 152 patients, an almost complete genome sequence of enteroviruses was obtained in 38% (58 patients), while real-time PCR confirmed the presence of enterovirus infection in all patients. The lower efficiency of genome-wide amplification in comparison with the original article [18] may be due to the lack of information about the viral load in the studied samples, RNA degradation, and differences in the amplification reagents used. The assembly revealed coinfection in 6 of the 58 patients. In patients with aseptic meningitis, the presence of two serotypes was established in 2 samples from CSF (E21/CVB5 and E11/CVB5), 2 samples from NSW (E6/CVA9), and in one patient, 3 serotypes were obtained (CSF - Coxsackievirus A9 and Echovirus E9/Coxsackievirus A9 in a sample from NSW), while the complete coding sequences of Coxsackievirus A9 from NSW and CSF were completely identical. The patient with HA was found to be coinfected with NSW CVB5/E6. The introduction of molecular genetic tests and sequencing schemes for genotyping enteroviruses has expanded our understanding of the epidemiology of enteroviruses and led to the discovery of coinfection. Coinfection is most often detected in patients with HFMD syndrome and can reach 34%, while simultaneous persistence of different serotypes can aggravate the severity of the infection and lead to atypical cases involving the respiratory and cardiovascular systems [40–42]. In the current study, we did not record any specific clinical course in patients with coinfections, which may be due to the limited sample size.

The most prevalent enterovirus genotype circulating in Astana during the seasonal rise of enterovirus infection in 2022 is CVA9, which was detected in 18 of 50 patients with aseptic meningitis and 6 of 8 patients with herpangina. Coxsackievirus A9, like many enteroviruses, exhibits a wide range of clinical manifestations, including aseptic meningitis (AM), hand, foot, and mouth disease (HFMD), acute flaccid paralysis (AFP), and persistent diarrhoea. Generalized febrile exanthema can cause pathologies in the placenta in pregnant women and severe generalized infections in newborns [43–46]. In some outbreaks of aseptic meningitis, CVA9 was the main serovariant in China [47], Canada [48], and South Africa [49]. CVA9 belongs to the virus serovars that were most often isolated in serous meningitis in Russia, while in 2022, there was an increase in the incidence of meningitis associated with CVA9 [50, 51]. CVA9 isolated in our study belongs to genotype I, which is mainly represented by Russian strains [23]. Kazakhstan and Russia have the longest land border in the world and close economic and cultural ties, which may facilitate the circulation of identical genotypes.

Coxsackievirus B5 in our study was the second most abundant enterovirus identified in 12 people with aseptic meningitis and in 2 patients with enterovirus herpangina. In global distribution, CVB5 is not a common serovariant in severe neurological complications, including acute flaccid paralysis, encephalitis, and aseptic meningitis [52]. However, in Poland [53] and China [24, 54, 55], CVB5 has been the predominant serovariant in several outbreaks in recent years. As shown in Farshadpour and Taherkhani [56], enterovirus was identified as a main cause of aseptic meningitis among studied population in Iran. Echovirus 30 was emerged as a predominant strain that caused aseptic meningitis in children in their first year of life [56]. Genotyping clustered the sequences of the current study into genotype D, which is predominantly represented by sequences from China. Given the migration pattern of CVB5 from Northeast China to Northwest China and East China to Northwest China [24], it is tempting to hypothesize a shift in the CVB5 circulation boundaries from China to Kazakhstan.

Echoviruses that were predominant in our study were E6 and E9. Echovirus 6 is one of the five enteroviruses that were diagnosed as the etiological agent of neurological complications, including acute flaccid paralysis (AFP), encephalitis, and aseptic meningitis [52, 57, 58]. In our case, E6 was detected in 8 patients with aseptic meningitis, of which only one in CSF and one case with herpangina, while coinfection with other enteroviruses was identified in 4 patients. High levels of E6 coinfection may promote recombination changes, requiring monitoring of the serovariant at the genetic level and tracking changes in virulence [59].

Nevertheless, official statistical data indicate a seasonal increase in enterovirus infections in 2022. Regular genotype studies in enterovirus infections will improve the control and differentiation of outbreaks from seasonal increases and assess the prevalence of dominant genotypes and the association of the clinical course with enterovirus genotypes in Kazakhstan. Analysis of global data shows that echovirus 30, echovirus 6, and echovirus 13 are most commonly detected in enterovirus meningitis [52]. Echoviruses also tend to predominate in meningitis cases recorded during endemic periods. A four-year follow-up in Poland shows the predominance of echovirus 6 and echovirus 30 in enterovirus meningitis cases [60]. In China, E6, E18, E11, E9, E30, and CVB5 were identified as the most common genotypes in aseptic meningitis between 2009 and 2018 [61–63]. In Latin American countries, aseptic meningitis is also usually dominated by echoviruses E30 and E6 [64]. In our case, the predominance of the CVA9 genotype is observed in aseptic meningitis and herpangina, which is usually characteristic of outbreaks [47–49]. Nevertheless, official statistical data indicate a seasonal increase in enterovirus infections in 2022. Regular genotype studies in enterovirus infections will improve the control and differentiation of outbreaks from seasonal increases and assess the prevalence of dominant genotypes and the association of the clinical course with enterovirus genotypes in Kazakhstan.

Current study had several limitations, such as the strategy of laboratory diagnosis protocols in hospital laboratories requires sending the samples for molecular detection analysis using RT-PCR, which in some cases is very time consuming, and doctors might have to wait for the results before the treatment. Reliable rapid diagnosis of enterovirus infections is essential in children; therefore, the use of sensitive screening tests for EV infection detection is important in clinical settings and hospitals. However, in most cases, symptoms are mild in patients' with enteroviral infections and require only supportive medications aimed at relieving the symptoms because it is challenging to distinguish between viral infections with similar symptoms based only on clinical representation. Given the prevalence of asymptomatic EV infections, merely detecting EV in a specimen does not necessarily indicate the cause of disease.

Identifying enterovirus serotypes is challenging and requires sequencing of the strains or sequencing of the VP1 gene, which is not available in some regions of the country. Genotyping can be more informative compared to serotyping, and utilizing the nucleotide sequence of VP1 can serve as a proxy for antigenic typing, aiding in distinguishing between enterovirus serotypes. This study aimed to explore the genetic diversity of enterovirus circulating in the area and to give a broader understanding of the processes underlying this transmission of enteroviral infection among children in Kazakhstan.

## 6. Conclusions

The reason for the seasonal rise in enterovirus infection in Astana in 2022 is eight serovars of group B enteroviruses; regular monitoring with genotyping is essential to understand any changes in their population structure. The central Eurasian location of Kazakhstan facilitates the circulation of both European and Asian genotypes of enteroviruses. The detection of coinfection in CSF and NSW, as well as the identification of different serotypes in the patients' CSF and NSW samples, suggests the possibility of misidentification of the etiological agent of aseptic meningitis during virological examination of NSW and stool samples. The introduction of NGS genotyping will expand knowledge about the possible coinfection of different enteroviruses and, consequently, recombination patterns in them.

## Figures and Tables

**Table 1:** Enterovirus results obtained in current study with metadata from patients.

#	Diagnosis new	Age	Sex	Date_hosp	Date_sample	Headache	Fever	Vomit	Weakness	Rush	Severity	Sample type	Sample ID	Serotype
1	Aseptic meningitis	5.0	F	9/11/2022	11.09.2022	Y	38	Y	Y	N	N	CSF	EV-105	CVA9
2	Aseptic meningitis	13.0	M	6/3/2022	03.06.2022	Y	38.5	Y	Y	N	N	CSF	EV-12	CVB5
3	Aseptic meningitis	5.0	M	6/1/2022	02.06.2022	Y	39	Y	Y	N	N	CSF	EV-14	E11
4	Aseptic meningitis	9.0	M	6/14/2022	14.06.2022	Y	37.5	Y	Y	N	N	CSF	EV-16	Echo21
5	Aseptic meningitis	7.0	M	6/14/2022	14.06.2022	Y	37.2	Y	Y	N	N	CSF1	EV-17 (vir1-vir2)	E11
												CSF2		Echo21
6	Aseptic meningitis	13.0	F	6/10/2022	12.06.2022	Y	38	Y	Y	N	N	CSF	EV-18	CVB5
7	Aseptic meningitis	5.0	F	6/12/2022	01.06.2022	Y		N	Y	N	N	CSF	EV-2	E9
8	Aseptic meningitis	10.0	M	6/9/2022	09.06.2022	Y	38	Y	Y	N	N	CSF1	EV-22 (vir1-vir2)	E11
												CSF2		CVB5
9	Aseptic meningitis	6.0	M	6/8/2022	08.06.2022	Y	38.2	Y	Y	N	N	CSF	EV-25	CVA9
10	Aseptic meningitis	8.0	M	6/15/2022	15.06.2022	Y	37.6	Y	Y	N	N	CSF	EV-28	E9
11	Aseptic meningitis	8.0	M	6/16/2022	16.06.2022	Y	39.9	Y	Y	N	N	CSF	EV-31	CVA9
12	Aseptic meningitis	8.0	M	6/15/2022	15.06.2022	Y	37	Y	Y	N	N	CSF	EV-32	E9
13	Aseptic meningitis	13.0	M	6/23/2022	23.06.2022	Y	37.5	Y	Y	N	N	CSF	EV-38	CVA9
14	Aseptic meningitis	4.0	F	6/16/2022	17.06.2022	Y	38.5	Y	Y	N	N	CSF	EV-39	E9
15	Aseptic meningitis	8.0	F	6/20/2022	20.06.2022	Y	37.8	Y	Y	N	N	CSF	EV-41	CVA9
16	Aseptic meningitis	6.0	M	6/20/2022	20.06.2022	Y	38.9	Y	Y	Y	N	CSF	EV-42	CVA9
17	Aseptic meningitis	9.0	F	6/27/2022	27.06.2022	Y	39	Y	Y	N	N	CSF	EV-45	CVA9
18	Aseptic meningitis	5.0	M	6/27/2022	27.06.2022	Y	38	Y	Y	N	N	CSF	EV-49	CVA9
19	Aseptic meningitis	12.0	M	6/29/2022	29.06.2022	Y	39	Y	Y	N	N	CSF	EV-50	CVB5
20	Aseptic meningitis	13.0	F	6/29/2022	29.06.2022	Y	38.4	Y	Y	N	N	CSF	EV-53	CVA9
21	Aseptic meningitis	8.0	M	5/29/2022	29.05.2022	Y	37.8	Y	Y	N	N	CSF	EV-6	CVA9
22	Aseptic meningitis	9.0	M	7/2/2022	02.07.2022	Y	39	Y	Y	N	N	CSF	EV-64	CVB5
23	Aseptic meningitis	7.0	F	7/10/2022	10.07.2022	Y	38.6	Y	Y	N	N	CSF	EV-69	CVB5
24	Aseptic meningitis	17.0	M	5/31/2022	31.05.2022	Y	39.1	Y	Y	N	N	CSF	EV-7	CVA9
25	Aseptic meningitis	12*π*	M	9/6/2022	06.09.2022	Y	38.3	Y	Y	N	N	CSF	EV-A1-2	CVB5
26	Aseptic meningitis	9*π*	F	6/24/2022	24.06.2022	Y	37.8	Y	Y	N	N	SWAB	EV-A2	E9
27	Aseptic meningitis	12*π*	M	9/18/2022	18.09.2022	Y	38	Y	Y	N	N	SWAB	EV-A2-2	CVB5
28	Aseptic meningitis	9.0	M	6/29/2022	28.06.2022	Y	37.2	Y	Y	N	N	SWAB	EV-A3	E25
29	Herpangina	6.0	F	7/5/2022	06.07.2022	N	38.5	Y	Y	Y	N	SWAB	EV-A4	CVA9
30	Aseptic meningitis	3.5	M	6/21/2022	23.06.2022	Y	38	Y	Y	N	N	SWAB	EV-B1	CVA9
31	Aseptic meningitis	4.0	F	9/10/20222	11.09.2022	Y	39	Y	Y	N	N	CSF	EV-B-2	CVB5
32	Aseptic meningitis	8.0	F	9/8/20222	08.09.2022	Y	38.5	Y	Y	N	N	SWAB	EV-B2-2	CVB3
33	Aseptic meningitis	9.0	M	6/28/20222	28.06.2022	Y	38.5	Y	Y	N	N	SWAB	EV-B3 (vir1-vir2)	E6
												SWAB		CVA9
34	Aseptic meningitis	10.0	M	7/9/20222	09.07.2022	Y	37.5	Y	Y	N	N	SWAB	EV-B4	E25
35	Aseptic meningitis	4.0	M	6/20/20222	20.06.2022	Y	37.8	Y	Y	N	N	SWAB	EV-C1	CVB5
36	Aseptic meningitis	7.0	M	7/14/20222	17/14/20222	Y	39	Y	Y	N	N	SWAB	EV-C1-2	E6
37	Herpangina	1.3	F	9/12/20222	13.09.2022	N	38.3	N	Y	Y	N	SWAB	EV-C2-2	CVA9
38	Aseptic meningitis	8.0	M	6/27/20222	27.06.2022	Y	38	Y	Y	N	N	SWAB	EV-C3	CVA9
39	Aseptic meningitis	5.0	F	7/17/20222	11.07.2022	Y	38.8	Y	Y	N	N	SWAB	EV-C4	E6
40	Herpangina	1.9	M	6/21/20222	22.06.2022	N	39	N	Y	N	N	SWAB	EV-D1	CVA9
41	Aseptic meningitis	5.0	F	7/15/20222	15.07.2022	Y		N	Y	N	N	SWAB	EV-D1-2	Echo21
42	Aseptic meningitis	4.0	M	6/23/20222	23.06.2022	Y	38.8	Y	Y	N	N	SWAB	EV-D2	E25
43	Herpangina	3.6	F	6/28/20222	29.06.2022	N	39	N	Y	N	N	SWAB	EV-D3	CVA9
44	Herpangina	6.0	M	7/12/20222	12.07.2022	Y	39	Y	Y	N	N	SWAB	EV-D4	CVA9
45	Aseptic meningitis	14.0	M	6/23/20222	23.06.2022	Y	37.5	Y	Y	N	N	SWAB	EV-E1	E9
46	Aseptic meningitis	9.0	M	9/23/20222	24.09.2022	Y	37.3	Y	Y	N	N	CSF	EV-E1-2	CVB5
47	Herpangina	2.3	M	6/23/20222	29.06.2022	N	39	N	Y	N	N	SWAB1	EV-E2 (vir1-vir2)	CVB5
	Herpangina	2.3	M	6/23/20222	29.06.2022	N	39	N	Y	N	N	SWAB2		E6
48	Aseptic meningitis	6.0	M	6/27/20222	27.06.2022	Y	38	Y	Y	N	N	SWAB	EV-E3	E6
49	Aseptic meningitis	13.0	M	7/13/20222	13.07.2022	Y	38	Y	Y	N	N	SWAB	EV-E4	E6
50	Aseptic meningitis	7.0	M	6/22/20222	23.06.2022	Y	38	Y	Y	N	N	SWAB	EV-F1	E6
51	Aseptic meningitis	5.0	M	9/9/20222	09.09.2022	Y	37.8	Y	Y	N	N	CSF	EV-F1-2	CVA9
52	Aseptic meningitis	2.4	M	6/23/20222	24.06.2022	N	39.6	N	Y	Y	N	SWAB	EV-F2	E9
53	Herpangina	15.0	M	6/28/20222	29.06.2022	N	38.5	Y	Y	N	N	SWAB	EV-F3	CVA9
54	Herpangina	1.2	F	6/22/20222	23.06.2022	Y	39.2	Y	Y	N	N	SWAB	EV-G1	CVB5
55	Aseptic meningitis	4.0	F	9/9/20222	09.09.2022	Y	39	Y	Y	N	N	CSF	EV-G1-2	E6
56	Aseptic meningitis	5.0	F	6/29/20222	29.06.2022	Y	40	Y	Y	N	N	SWAB1	EV-G3 (vir1-vir2)	E6
												SWAB2		CVA9
57	Aseptic meningitis	13.0	M	6/23/20222	23.06.2022	Y	37.5	Y	Y	N	N	SWAB1	EV-H1 (vir1-vir2)/E	E9
												SWAB2		CVA9
												CSF		CVA9
58	Aseptic meningitis	12.0	F	7/4/20222	05.07.2022	Y	39	Y	Y	N	N	SWAB	EV-H3	CVA9

## Data Availability

Sequences obtained within the current study were submitted to GenBank under OR678399-OR678463 accession numbers.

## Supplementary Materials

Supplementary Materials Figures S1–S8 represent maximum likelihood (ML) phylogenetic trees reconstructed for specific human enterovirus B serotypes CVA9, CVB5, CVB3, E6, E9, E11, E21, and E25, respectively, using the whole VP1 gene sequences of collected from available worldwide sequences (of the serogroup) and from infected patients in Kazakhstan. Within each serotype, the genotypes' classification is shown based on within (<15%) and between clades' diversity (>15%); bootstrap values >50% shown as blue circles. Tables S1-S2 demonstrate the estimates of evolutionary divergence between the sequences of serotype E21 and E25, respectively. The number of base substitutions per site from between sequences is shown. A lower triangle shows between clades' (genotype groups) divergence in nucleotides. Diagonals show within clades' (genotype groups) divergence in nucleotides within each serotype. Tables S3–S5 represent estimates of pairwise evolutionary divergence between sequences within serotypes CVA9, CVB5, and E6, respectively. The number of base substitutions per site between sequences is shown.
