# Supplementary material for: Genetic Diversity of Human Enterovirus in Kazakhstan, during 2022
Source: Int J Microbiol. 2024 Aug 26;2024:7796913. doi: 10.1155/2024/7796913 (PMC11368554; doi:10.1155/2024/7796913)
Supplement: Supplementary Materials — Figures S1–S8 represent maximum likelihood (ML) phylogenetic trees reconstructed for specific human enterovirus B serotypes CVA9, CVB5, CVB3, E6, E9, E11, E21, and E25, respectively, using the whole VP1 gene sequences of collected from available worldwide sequences (of the serogroup) and from infected patients in Kazakhstan. Within each serotype, the genotypes' classification is shown based on within (<15%) and between clades' diversity (>15%); bootstrap values >50% shown as blue circles. Tables S1-S2 demonstrate the estimates of evolutionary divergence between the sequences of serotype E21 and E25, respectively. The number of base substitutions per site from between sequences is shown. A lower triangle shows between clades' (genotype groups) divergence in nucleotides. Diagonals show within clades' (genotype groups) divergence in nucleotides within each serotype. Tables S3–S5 represent estimates of pairwise evolutionary divergence between sequences within serotypes CVA9, CVB5, and E6, respectively. The number of base substitutions per site between sequences is shown. [file 7796913.f1.zip › Tables_S3_Estimates of pairwise evolutionary divergence between sequences within serotypes CVA9, CVB5 and E6, respectively.pdf]

Table S3. Estimates of pairwise evolutionary divergence between sequences of serotype CVA9 (Figure S1). The number of base substitutions per site from between sequences are shown. Lower triangle shows between sequence pairs divergence in nucleotides.

Sequences IDs	8509	8615	10158	9763	10198	9321	9497	9896	9711	9725	10143	10116	10278	9771	10180	10189	10232	9896	10513	10957	10564	9057	14354	14196	14313
**8509**																									
**8615**	0,003																								
**10158**	0,102	0,101																							
**9763**	0,073	0,073	0,044																						
**10198**	0,102	0,101	0,001	0,043																					
**9321**	0,015	0,014	0,099	0,071	0,099																				
**9497**	0,101	0,100	0,003	0,043	0,002	0,098																			
**9896**	0,101	0,100	0,028	0,043	0,027	0,098	0,027																		
**9711**	0,004	0,003	0,102	0,074	0,102	0,016	0,101	0,101																	
**9725**	0,098	0,097	0,016	0,041	0,015	0,096	0,015	0,026	0,098																
**10143**	0,074	0,073	0,046	0,026	0,045	0,072	0,045	0,048	0,074	0,044															
**10116**	0,101	0,100	0,001	0,043	0,000	0,098	0,002	0,027	0,101	0,015	0,045														
**10278**	0,074	0,073	0,048	0,027	0,047	0,073	0,046	0,050	0,075	0,046	0,010	0,046													
**9771**	0,004	0,002	0,101	0,073	0,101	0,015	0,100	0,100	0,004	0,097	0,073	0,100	0,074												
**10180**	0,083	0,082	0,061	0,051	0,060	0,081	0,060	0,060	0,083	0,058	0,037	0,060	0,042	0,083											
**10189**	0,075	0,074	0,046	0,026	0,045	0,073	0,045	0,048	0,075	0,044	0,002	0,045	0,009	0,074	0,037										
**10232**	0,076	0,075	0,047	0,027	0,046	0,074	0,046	0,049	0,077	0,045	0,003	0,046	0,011	0,076	0,038	0,001									
**9896**	0,101	0,100	0,028	0,043	0,027	0,098	0,027	0,000	0,101	0,026	0,048	0,027	0,050	0,099	0,059	0,047	0,048								
**10513**	0,014	0,013	0,097	0,069	0,097	0,011	0,096	0,096	0,014	0,094	0,069	0,096	0,070	0,013	0,079	0,070	0,071	0,096							
**10957**	0,074	0,073	0,047	0,026	0,046	0,072	0,046	0,048	0,074	0,045	0,009	0,046	0,008	0,073	0,042	0,008	0,010	0,048	0,069						
**10564**	0,074	0,073	0,046	0,026	0,046	0,072	0,045	0,048	0,074	0,044	0,000	0,045	0,010	0,074	0,037	0,002	0,003	0,048	0,069	0,009					
**9057**	0,005	0,004	0,101	0,073	0,101	0,014	0,100	0,100	0,005	0,097	0,072	0,100	0,074	0,005	0,082	0,073	0,075	0,100	0,012	0,072	0,072				
**14354**	0,078	0,076	0,081	0,059	0,081	0,076	0,080	0,083	0,079	0,080	0,061	0,081	0,061	0,078	0,066	0,061	0,063	0,083	0,074	0,061	0,061	0,077			
**14196**	0,101	0,100	0,009	0,044	0,009	0,098	0,009	0,029	0,101	0,017	0,045	0,008	0,048	0,100	0,060	0,045	0,046	0,029	0,095	0,047	0,046	0,100	0,081		
**14313**	0,074	0,073	0,046	0,026	0,046	0,072	0,045	0,048	0,074	0,044	0,000	0,045	0,010	0,074	0,037	0,002	0,003	0,048	0,069	0,009	0,000	0,072	0,061	0,046	
