# Supplementary material for: Genetic Diversity of Human Enterovirus in Kazakhstan, during 2022
Source: Int J Microbiol. 2024 Aug 26;2024:7796913. doi: 10.1155/2024/7796913 (PMC11368554; doi:10.1155/2024/7796913)
Supplement: Supplementary Materials — Figures S1–S8 represent maximum likelihood (ML) phylogenetic trees reconstructed for specific human enterovirus B serotypes CVA9, CVB5, CVB3, E6, E9, E11, E21, and E25, respectively, using the whole VP1 gene sequences of collected from available worldwide sequences (of the serogroup) and from infected patients in Kazakhstan. Within each serotype, the genotypes' classification is shown based on within (<15%) and between clades' diversity (>15%); bootstrap values >50% shown as blue circles. Tables S1-S2 demonstrate the estimates of evolutionary divergence between the sequences of serotype E21 and E25, respectively. The number of base substitutions per site from between sequences is shown. A lower triangle shows between clades' (genotype groups) divergence in nucleotides. Diagonals show within clades' (genotype groups) divergence in nucleotides within each serotype. Tables S3–S5 represent estimates of pairwise evolutionary divergence between sequences within serotypes CVA9, CVB5, and E6, respectively. The number of base substitutions per site between sequences is shown. [file 7796913.f1.zip › Tables_S5_Estimates of pairwise evolutionary divergence between sequences within serotypes CVA9, CVB5 and E6, respectively.pdf]

Table S5. Estimates of pairwise evolutionary divergence between sequences of serotype E6 (Figure S4). The number of base substitutions per site from between sequences are shown. Lower triangle shows between sequence pairs divergence in nucleotides.

Sequences IDs	10180	9876	9920	10142	10232	11079	10887	10993	14161
**10180**									
**9876**	0,016								
**9920**	0,001	0,017							
**10142**	0,001	0,017	0,002						
**10232**	0,016	0,000	0,017	0,017					
**11079**	0,014	0,017	0,015	0,014	0,017				
**10887**	0,015	0,016	0,016	0,016	0,016	0,015			
**10993**	0,015	0,010	0,016	0,016	0,010	0,015	0,014		
**14161**	0,017	0,012	0,018	0,018	0,012	0,017	0,016	0,008	
